# Tissue-Dependent Tumor Microenvironments and Their Impact on Immunotherapy Responses

**DOI:** 10.3389/fimmu.2018.00070

**Published:** 2018-01-31

**Authors:** Amanda J. Oliver, Peter K. H. Lau, Ashleigh S. Unsworth, Sherene Loi, Phillip K. Darcy, Michael H. Kershaw, Clare Y. Slaney

**Affiliations:** ^1^Cancer Immunology Program, Peter MacCallum Cancer Center, Melbourne, VIC, Australia; ^2^Sir Peter MacCallum Department of Oncology, University of Melbourne, Parkville, VIC, Australia

**Keywords:** tumor microenvironment, tissue-specific microenvironment, immunotherapy, immunosuppression, anticancer therapy

## Abstract

Recent advances in cancer immunology have led to a better understanding of the role of the tumor microenvironment (TME) in tumor initiation, progression, and metastasis. Tumors can occur at many locations within the body and coevolution between malignant tumor cells and non-malignant cells sculpts the TME at these sites. It has become increasingly clear that there are specific differences of the TMEs at different anatomical locations, and these tissue-specific TMEs regulate tumor growth, determine metastatic progression, and impact on the outcome of therapy responses. Herein, we review the scientific advances in understanding tissue-specific TMEs, discuss their impact on immunotherapeutic response, and assess the current clinical knowledge in this emerging field. A deeper understanding of the tissue-specific TME will help to develop effective immunotherapies against tumors and their metastases and assist in predicting clinical outcomes.

## Introduction

Tumor cells do not grow in isolation, but exist in a complex tumor microenvironment (TME), which the tumor cells depend upon for growth and metastasis. The TME comprises cells of hematopoietic origin (lymphocytes and myeloid cells), mesenchymal origin (fibroblasts, myofibroblasts, mesenchymal stem cells, adipocytes, and endothelial cells), and the extracellular matrix (ECM) (1). The components of the TME are manipulated by tumor cells and participate in tumor progression throughout all stages of tumorigenesis (1).

Tumors can arise in, and metastasize to, various tissues. Clear evidence suggests that the tissue of tumor growth influences the TME composition (2, 3). These tissue-specific TMEs regulate tumor growth, determine metastatic progression, and impact the outcome of therapy responses. In this review, we discuss tissue-specific differences in the TME and its impact on therapeutic response. We propose that understanding such differences is important for the development of effective immunotherapies against tumors and their metastases.

### The Immunosuppressive TME and Its Impact on Therapeutic Response

**Figure 1:**
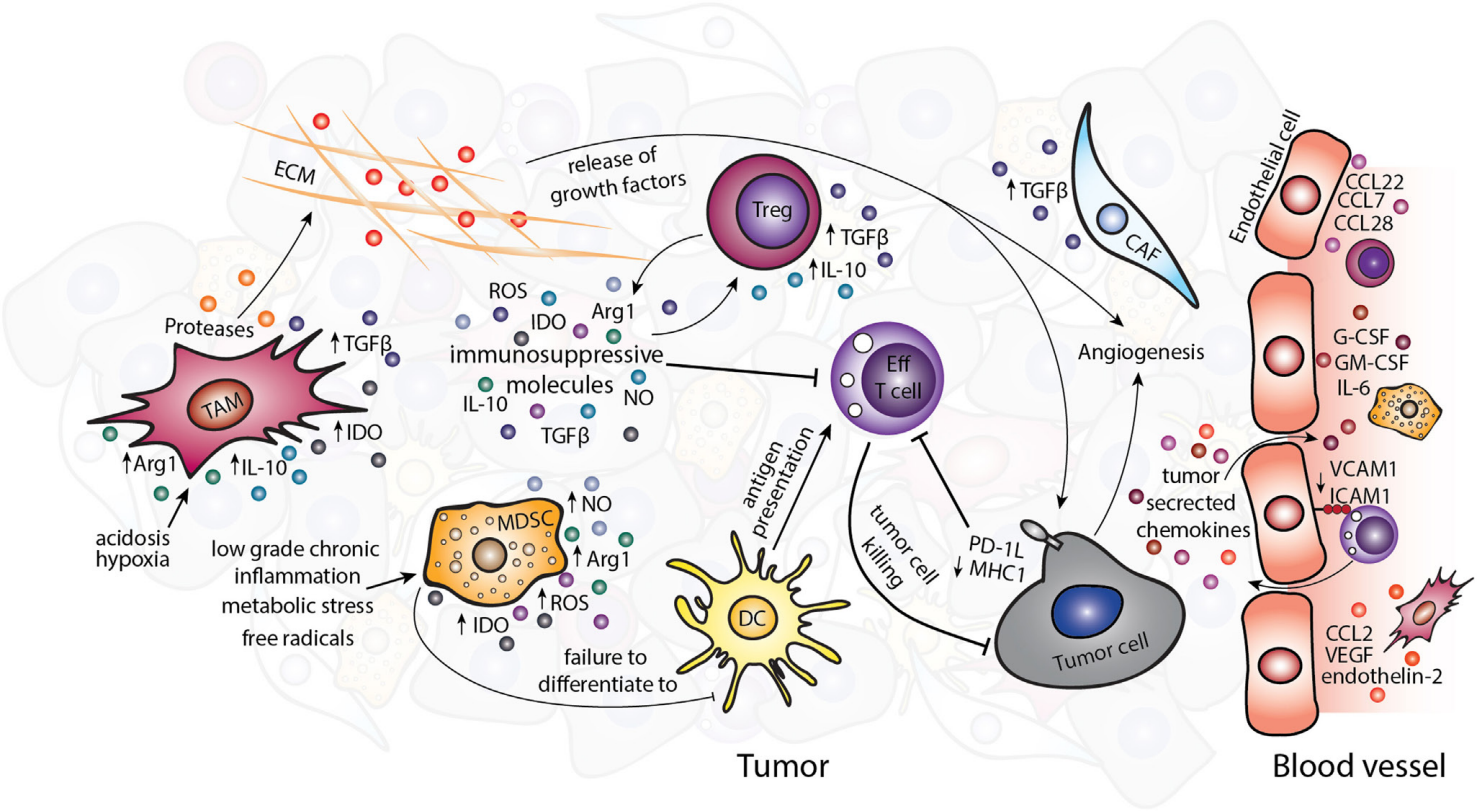
The immunosuppressive tumor microenvironment. Various immunosuppressive cell types exist within the TME including TAMs, MDSCs, Tregs, and CAFs. Tumor-derived chemokines and the metabolic tumor environment recruit or polarize these cells to a protumor, immunosuppressive phenotype. Suppression of the antitumor immune response occurs partly through the release of immunosuppressive molecules such as TGF-β, IL-10, IDO, Arg1, ROS, and NO. Abbreviations: Arg1, arginase1; CAFs, cancer-associated fibroblasts; DC, dendritic cell; ECM, extracellular matrix; Eff T cell, effector T cell; IDO, idoleamine 2,3-dioxygenase; MDSCs, myeloid-derived suppressor cells; ROS, reactive oxygen species; NO, nitric oxide; TAMs, tumor-associated macrophages; Tregs, regulatory T cells.

Avoiding immune destruction is an emerging hallmark of cancer (4). The established TME contains cell types that can contribute to immune evasion by inhibiting effective antitumor response of effector cells (Figure 1). The immunosuppressive and other protumorigenic cell types within the TME have been reviewed in detail elsewhere (1, 5). Briefly, as shown in Figure 1, immunosuppressive cell types such as regulatory T cells (Tregs), myeloid-derived suppressor cells (MDSCs), and tumor-associated macrophages (TAMs) can be present within the TME. These cells can express immunomodulatory factors such as interleukin (IL)-4, IL-10, IL-13, and arginase1 (Arg1), which suppress or reprogram the antitumor immune response (6–8), for example, by depletion of the essential amino acid arginine or the skewing of immunity toward a Th2-type response ill-suited to tumor cell destruction. Depleting these immunosuppressive cells in mouse models of cancer can reduce tumor growth and progression (9–11), and infiltration of these cells in human tumors has been associated with poor prognosis (12–17).

Non-immune cells of the TME also contribute to enhancing tumorigenesis and can directly influence the antitumor immune response. Cancer-associated fibroblasts (CAFs) can secrete protumorigenic molecules including mitogenic growth factors, pro-angiogenic factors, and TGF-β, which alter the TME and support cancer progression (18). The chaotic tumor vasculature that comprises endothelial cells and pericytes is usually leakier than normal vasculature and is therefore unable to support efficient trafficking of cytotoxic immune cells to the tumor (19, 20). The abnormalities of blood vessels have been identified in a number of tumor types in murine models, such as spontaneous RIP-Tag2 pancreatic islet tumors, MCA-IV mammary carcinomas, Lewis lung carcinomas (21), 4T1 mammary carcinoma (22), and B16F10 melanoma (23), although studies directly comparing different tumor types and subtypes, especially in human cancers are lacking. The expression of pro-angiogenic signals in the TME, such as stromal-derived factor-1, thrombospondin, and matrix metalloproteases secreted by CAFs (24) and VEGF by TAMs (25) can further contribute to altered tumor vasculature (26). The non-cellular ECM that plays an important part in tissue homeostasis is also altered in tumors by the imbalance between ECM synthesis and secretion and changes in the levels of matrix-remodeling enzymes (27). The altered ECM results in changes to the tissue architecture and release of soluble molecules and growth factors. These changes further propagate the TME partly *via* influencing the actions of immune cells (Figure 1) (28, 29).

Immunotherapeutics that aim to alter the immune TME to target cancers have revolutionized the treatment of cancer. Attention has recently focused on two classes of immunotherapies, including immune checkpoint inhibitors (ICI) and adoptive cellular transfer (ACT). Melanoma has served as the test bed for ICI with the initial development of anti-CTLA-4 antibodies (ipilimumab) (30) and more recently antibodies that inhibit the programmed death-1 axis (e.g., nivolumab, pembrolizumab) (31, 32). The objective response rates were 43.7 and 19% in metastatic melanoma patients treated with nivolumab and ipilimumab alone, and the combination of nivolumab and ipilimumab resulted in a much higher response rate (57.6%) (26). Besides, ICI have also established efficacy in a range of other solid tumors such as non-small cell lung cancer (NSCLC) (33, 34) and renal cell carcinoma (35). In these trials, the objective response rates were 14.5% (33) and 44.8% for patients with refractory/advanced NSCLC (34), and 20–22% for metastatic renal cell carcinoma patients (35). Recent developments of chimeric antigen receptor-T cells in CD19 hematological malignancies have led to high complete response rates and durable regressions in both lymphoma and leukemia (36, 37) and has generated some promising results in solid cancers in small studies (38). Despite these successes, not all patients obtain clinical benefit, which is often attributable to *de novo* resistance mediated by TME.

A role for the TME in resistance to anticancer immunotherapies has been established. Various cell subsets that contribute to an immunosuppressive TME are associated with reduced therapeutic efficacy. Higher numbers of MDSCs correlate with poor response to various immunotherapies including immune checkpoint blockade (39), ACT (40), and dendritic cell (DC) vaccination (41). The ratio of effector T cells (Eff T cells) to Tregs is associated with response to anti-CTLA-4 checkpoint blockade therapy, where higher Tregs are associated with decreased efficacy (42, 43). Blocking the recruitment of TAMs using anti-CSF-1R antibodies is synergistic with ACT and checkpoint blockade therapy, indicating that TAMs have a crucial role in mediating response to immunotherapy (44, 45). The influence of the TME on therapeutic response is not restricted to immunotherapies and has also been shown for various anticancer therapies including those directly targeting malignant cells such as chemotherapy (46–49). Thus, the TME has a notable impact on the outcome of anticancer therapeutics and its consideration is essential for effective immunotherapies.

In summary, there is a clear role for the TME in modulating responses to tumor and stromal targeted anticancer therapies. The complexity and adaptability of the TME during tumor development and in response to various treatments remains to be properly characterized and is a challenge within itself. Our current knowledge of the progression and sculpting of the TME is somewhat limited; however, there is clear evidence for tissue-specific tumor development.

### Tissue-Specific TMEs

There is clear evidence that tumor initiation and metastasis is tissue specific. Cancer cells arising from the same organ or tissue often share specific driver mutations (50). In the case of familial cancers, inherited mutations in driver genes cause cancer in specific organs such as BRCA1 and BRCA2 in hereditary breast and ovarian cancer. The simplest explanation for this tissue-specific tumorigenesis would be that these mutated genes are only expressed in the tissues where the tumors commonly develop. However, this is not the case, as many driver genes are expressed in various tissues that do not form tumors from mutations in these genes. Instead, tissue-specific tumorigenesis can be explained by a multitude of factors (3). One of these is the likely presence of various cell types within the tissue microenvironment that is dependent on the anatomical location. For example, resident myofibroblast-like stellate cells within the liver and pancreas are pathogenic drivers of fibrosis and can promote tumor development (51). In addition, different cancer types tend to colonize specific organs, known as the seed and soil hypothesis or organotropism (52, 53). As a result of tumor-secreted factors and tumor-shed extracellular vesicles, the tissue microenvironment of metastatic sites is altered to form a premetastatic niche (54). This is similar to the manipulation of local non-malignant cells to form the primary TME as mentioned previously. Thus, the tissue of origin, including the non-malignant cell types within, is a specific regulator of malignant transformation and metastatic colonization.

Both preclinical and clinical evidence indicates the tissue of tumor growth as an influential factor in the established TME. Although some effort has been made to understand how the tissue-specific microenvironment interacts with tumors at different sites, it is difficult to eliminate the effect of tumor cell heterogeneity due to the genetic heterogeneity of tumors (55). Only a few groups of investigators have used preclinical murine models of cancer with implantation of genetically identical tumor cell lines at various anatomical sites to eliminate tumor cell dependent heterogeneity. Such studies have shown that genetically identical tumors growing at different anatomical sites have site-specific transcript, protein, and metabolite profiles. For example, in murine models of pancreatic cancer using various cell lines (CD18/HPAF, FG, L3.3, L3.6pl, and BxPC3), multiple studies have shown that orthotopic or SC implanted tumors of the same cell line have different gene expression profiles (56–58). Analysis of RNA expression profiles in orthotopic tumors has shown elevated expression of known pancreatic cancer-associated genes such as MUC4 and TGFβ2. In separate studies, comparison of SC and orthotopic renal cell carcinoma (SN12C and SN12PM6) or orthotopic prostate cancer (PC-3M) showed decreased mRNA and protein expression of basic fibroblast growth factor in SC tumors (59, 60), which is known to promote angiogenesis. In the PC-3M prostate cancer model, orthotopic tumors expressed lower levels of other protumorigenic transcripts including the ones encoding EGFR, mdr-1, collagenase type IV, and IL-8 compared with SC tumors (60). Similarly, A375P and A375SM melanoma cells growing subcutaneously had higher expression of IL-8 by northern blot and IHC compared with melanoma cells growing in the lung (intermediate IL-8) and liver (low IL-8) (61). Recently, Zhan et al. performed a metabolomics study of pancreatic ductal adenocarcinoma cell lines (Panc-1 and BxPC-3) growing SC or orthotopically using ^1^H NMR spectroscopy. Clear differences in metabolites in the tumors, but not in serum, were detected between mice with SC and orthotopic tumors. Notably, the orthotopic tumors had higher levels of adenosine (an immunosuppressive metabolite) compared with SC tumors (62). Thus, current evidence in the field suggests the tissue of tumor growth can influence the molecular composition of tumors including RNA, protein, and metabolites. Furthermore, comparisons between orthotopic and SC tumors suggest that the TME of orthotopic tumors are more immunosuppressive and protumorigenic.

The cellular composition of the TME can also vary depending on the tissue of tumor growth. For example, in a murine model of breast cancer, the immune cell profile was compared between 4T1 tumors growing SC or intratibially (63). FACS analysis in this model revealed differences in the proportions of macrophages, DCs, CD8^+^, and CD4^+^ T cells in the tibia and under the skin of mice with tumors growing at these sites. Interestingly, the site of tumor growth also affected the immune cell populations in the spleen, as the mice bearing SC tumors displayed a significant decrease in T cells in their spleens compared with mice bearing tumors in the tibia. Similar observations have been reported in human cancers. An elegant study investigating multiple metastases in a patient with high-grade serous ovarian adenocarcinoma showed multiple distinct tumor immune microenvironments coexisted within the same patient. The immune infiltration and activation of the tumors assessed by IHC and RNAseq of immune-related genes were different in each tumor. Tumors that responded to chemotherapy were heavily infiltrated with Eff T cells, while the stable tumors had a lower level of T cell infiltration and the non-responding tumors lacked immune cell infiltration (64). Although detailed mechanisms remains unclear, these findings provide evidence that the local TME can alter immune infiltrates.

### Impact of Tissue-Specific TME on the Therapeutic Response

As discussed in the previous section, tumors growing at different anatomical sites have distinct TMEs. When tumors are present at different sites, these tissue-specific TMEs can influence the response to therapy at their own niche. The tissue-dependent difference in therapeutic response is most obvious in the field of immunotherapy.

Recent preclinical studies using immunotherapies to target tumors growing at different anatomical locations clearly demonstrated that the site of tumor growth could dictate the response to anticancer therapies. Our laboratory has demonstrated SC tumors are more responsive than visceral tumors to trimAb immunotherapy (anti-DR5, anti-CD40, and anti-4-1BB) in multiple murine tumor models (2). In this work, established SC tumors could be eradicated in mice using trimAb. However, the antitumor response to trimAb was found to be greatly reduced in orthotopic tumors compared with SC tumors, despite tumors in the two locations being of similar size. The dramatic difference in response was not due to the malignant cells, as tumor cells isolated from Renca SC and orthotopic tumors showed similar key characteristics, including major histocompatibility complex I and DR5 expression by FACS. When these re-isolated tumor cells were injected back into the same or opposite sites, the same site-specific response to trimAb was observed, regardless of where the tumor cells were isolated from. Comparison of immune infiltrates of orthotopic or SC Renca tumors by FACS revealed an increase in F4/80^high^CD206^+^ cells, which identifies the immunosuppressive M2 macrophages/TAMs. Furthermore, abolishing factors important for recruitment and differentiation of TAMs such as CCL2 and IL-13, improved the response of orthotopic Renca tumors indicating that this subset was partially responsible for the reduced efficacy to trimAb.

Tissue-specific responses to other immunotherapies have also been reported using other preclinical models. The response of TC-1 tumor stably expressing HPV16-E7 to a vaccine was dependent on the site of tumor implantation (65). The vaccine consists of mRNA encoding the HPV16-E7 oncoprotein together with TriMix, an mRNA-based vaccine encoding for CD40 ligand, constitutively active toll-like receptor 4 and CD70. The tumor cells implanted SC had the strongest response to E7-TriMix, with a less impressive response of tumors of the lung and a further reduced response when tumor cells were implanted into the genital tract. While the percentage by FACS of Tregs in SC tumors were dramatically decreased by vaccination, Tregs were only slightly decreased in the lung and unaffected in the genital tract tumors. In addition, genital tract tumors had a much higher percentages of both granulocytic and monocytic MDSCs compared with other tumors. The proportion of MDSCs did not decrease upon E7-TriMix treatment in the genital tract tumors as observed in the subcutaneous and lung models. In a colorectal cancer model using CT26 cells, orthotopic colon tumors had a higher infiltrate of T cells, B cells, and natural killer (NK) cells, but lower (CD11b^+^CD11c^−^) Ly6G^+^ or Ly6C^+^ myeloid cells compared with SC tumors. In this model, orthotopic tumors showed increased response to combination checkpoint blockade therapies (anti-CTLA-4 and anti-PD1) than the SC tumors (66). The tumor location-dependent difference in cellular responses to immunotherapy was also observed in a murine melanoma model (67). This study demonstrated that the recruitment of Ly6C^+^ monocytes from the blood was essential for antibody-dependent tumor cell killing of melanoma in the skin but not in the lung. It was proposed that the local tissue TME determined which immune populations contribute to the antitumor antibody activity and the therapeutic response.

These recent studies utilizing preclinical models treated with various immunotherapies provide evidence for the influence of tissue-specific microenvironments on immunotherapeutic response. Logically, the data suggest an association between immunosuppressive TMEs and reduced response to immunotherapies. Despite these studies, there is a requirement for further characterization of tissue-specific TMEs and response to immunotherapies and how this relates to human cancer. Although injection of genetically identical tumor lines eliminates the variable of tumor cell genetic heterogeneity in these models, there are limitations in applicability to human cancers. Notably, tumors in these models are established rapidly and the sculpting of the TME may differ from human tumors which could take much longer to establish. Despite this, there are clear correlations between tumors at certain sites and immunotherapy responses in human cancers.

A common clinical problem with advanced cancer patients is the differential response to systemic treatment where some lesions may be less responsive to therapy compared with other anatomical sites. While this may be representative of tumor heterogeneity, the local TME is likely to play a role. Survival patterns of patients with metastatic melanoma, a highly immunogenic cancer, can be dependent on anatomical sites of disease (68). In keeping with the preclinical models described above, patients with subcutaneous, lymph node or skin metastases exhibited better survival outcomes than those with lung or other non-pulmonary visceral metastases in an era without effective systemic treatment for melanoma (68). Furthermore, the response rate to high dose IL-2, a treatment reserved in only specialized melanoma centers, was approximately 50% in patients with subcutaneous metastases only compared with 13% with visceral metastases (69). Accordingly, ICI exhibit differential response rates at different anatomical sites, favoring patients with subcutaneous and lung sites. Retrospective analyses of anti-PD1-treated patients with advanced melanoma (70–72) and NSCLC (73) displayed poorer survival outcomes in the presence of liver metastases compared with other visceral sites such as lung. This observation was corroborated where the best objective response rates to pembrolizumab in melanoma patients with or without liver metastases was 33.3 and 71.4%, and in NSCLC 28.6 and 56.7%, respectively (72). Median progression-free survival of melanoma patients with liver metastases was poor at 2.7 months compared with 18.5 months in those without hepatic involvement. Moreover, CD8^+^ T cell density at the tumor margin, a key biomarker of response to anti-PD1 antibodies (74), was significantly lower in the liver metastases cohort compared with those without liver metastases (72). Similar observations have also been reported in breast cancer patients. In a cohort of metastatic triple-negative breast cancer patients treated with anti-PD1, the level of TILs and response to therapy varied significantly depending on metastases location (75). The presence of lymph node metastases was strongly associated with better response compared with metastases in other organs, such as the liver. Collectively, these studies are consistent with preclinical evidence supporting the role for tissue-specific TMEs in mediating immunotherapeutic responses. Regardless of cancer type, liver metastases overall had reduced response to ICI compared with metastases at other sites. Accordingly, characterization of the liver-specific TME should be of particular focus in subsequent studies.

### Cross Talk between Tumors in Different Tissues of the Body

Tumors can present simultaneously in different organs within the same patient either by metastatic growth or bilateral cancers. As previously discussed, the tissue-specific TME influences response to immunotherapy, with tumors in certain sites being more responsive than others. Recent publications have investigated the potential for such tumors to influence each other when present simultaneously. A study in our laboratory showed in mice that growth of a concomitant therapy-resistant tumor decreases efficacy of previously responsive tumors to immunotherapy (76). This was shown for SC Renca tumors when orthotopic kidney tumors were present in the same mice. The same was not observed when duplicate SC tumors were present simultaneously. The TME of SC tumors with a concomitant kidney tumor resembled the immunosuppressive TME previously observed in the kidney tumor model. This included an increase in the F4/80^high^CD206^+^ macrophages and a reduced Eff T and NK cell profile determined by FACS and analysis of immune-related gene expression of tumors. Blocking trafficking (with anti-CCL2 antibody) or depleting (with clodrolip) macrophages improved the effect of immunotherapy on these SC tumors, suggesting that immunosuppressive cells within a resistant tumor can migrate to responsive tumors and inhibit response to therapy.

Potential cross talk between tumors located at different sites has also been observed in humans. In metastatic melanoma, cutaneous/SC metastases with concomitant visceral metastases had a lower objective response rate (14%) to IL-2-based therapy compared with patients who had cutaneous/SC metastases alone (50%) (69). Presented at the 2017 ASCO meeting, Lee et al. from the University of California (77) reported that melanoma patients with additional liver metastases had a lower percentage of CD8^+^ Eff T cells but a higher percentage of CTLA-4^+^PD1^+^CD8^+^ activated-exhausted T-cells within the tumor-infiltrating lymphocytes and this was associated with a decreased response to PD1 blockade. They also investigated these findings in a murine model of B16 melanoma cells implanted SC and into the liver simultaneously or alone. Mice that had both SC and liver tumors had increased tumor growth compared with mice with SC tumors alone and had reduced response to anti-PD1 therapy, as seen in humans. Interestingly, presence of lung metastases or the implantation of unrelated MC38 liver tumors to the SC B16 tumor-bearing mice did not alter the SC tumor growth. This report indicated that liver metastases could cross talk with melanoma in the skin and lead to reduced Eff T cell responses and reduced response to PD1 blockade. These findings have important implications for directing treatment strategies especially since patients with multiple tumors are often much further advanced and harder to treat. Possibly removal or eradication of immunotherapy resistant tumors followed by administration of immunotherapy could benefit patient outcomes. Robust characterization of this cross talk is required to guide clinical decisions and treatment regimens.

## Concluding Remarks

**Figure 2:**
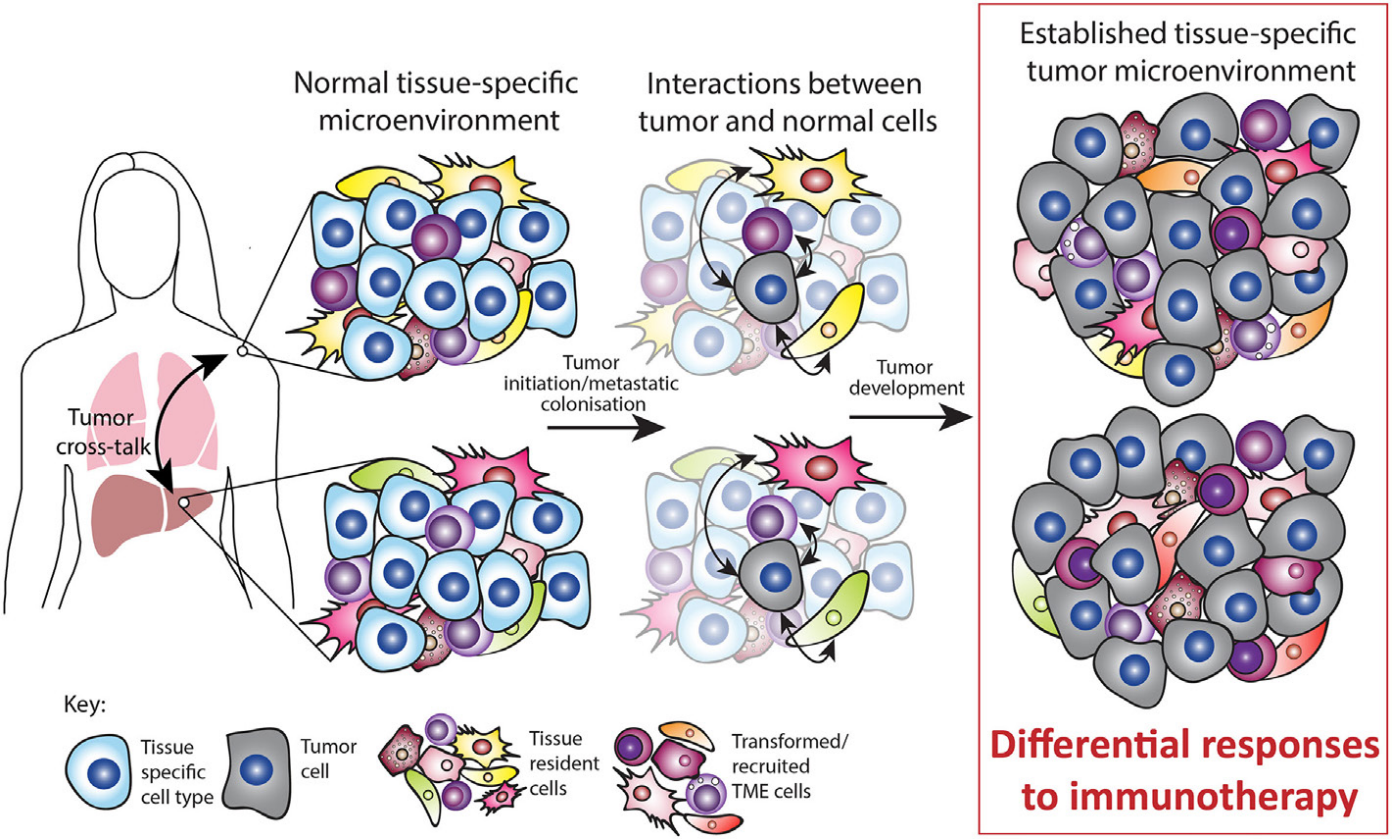
Tissue-specific tumor microenvironment (TME). Tumors can occur at various sites in the body and often occur simultaneously, for example, by metastatic growth. The normal tissue-specific microenvironment consists of both tissue-specific cell types and tissue-resident cell types such as immune, mesenchymal, and endothelial cells. Upon tumor initiation or metastatic colonization, interactions occur between tumor and normal cells. During tumor development, these interactions are partly responsible for the established TME. Both preclinical and clinical studies suggest that the tissue-specific TME mediates the response to immunotherapy. In addition, tumors occurring simultaneously within different TMEs can cross talk and influence each other. Thus, the normal tissue plays a major role in sculpting the established TME and this ultimately impacts on the response to immunotherapy.

Despite traditional focus on the malignant cells, non-malignant cells within the TME play an important role in tumor growth, progression, and response to therapy. As highlighted in this review, there is an emerging role for the tissue of tumor growth on the TME composition and response to immunotherapies. A number of recent studies suggest that tumor growth in different tissues promotes the development of tissue-specific TMEs and that this is an influential factor for therapy responses (Figure 2). Furthermore, emerging data suggest that tumors with disparate TMEs and therapy responses can cross talk and influence each other. As such further studies are required to firmly establish a conclusive role for tissue-specific TMEs in various contexts. A deeper understanding of these unique organ-specific mechanisms of resistance may allow personalized approaches to immunotherapy. With the plethora of novel immunotherapy combinations in development that target other immune checkpoints (e.g., LAG-3, TIM-3), cytokines (e.g., TGFβ), oncolytic viruses, and other immunosuppressive mediators (e.g., IDO, adenosine) these new agents may also have differential activity by organ site. Hence, tailoring novel immunotherapy combinations depending on the organ-specific TME may improve therapeutic benefit, particularly in metastatic disease.

However, characterization of the tissue-specific differences in human tumors poses both technical and investigational challenges. Due to tumor genetic heterogeneity, it is difficult to distinguish the influence of the organ microenvironment versus the cancer type and genetic mutations. Although cancers of the same type metastasize to different organs, the genetic mutations and phenotype of these disseminated cancer cells can differ from the primary cancer (55). Thus, studying tissue-specific differences within the TME is complicated by tumor genetic heterogeneity in the human setting. In addition, obtaining samples from multiple visceral metastatic sites can be technically challenging and is acceptable to only the most willing patients. Murine models may therefore be insightful, with orthotopic tumors displaying variations in their TME and response to therapy compared with subcutaneous tumors. Given murine subcutaneous tumors respond to therapy much better than orthotopic tumors, the latter are likely to provide better predictors of therapeutic efficacy in primary tumors and permit successful translation into the clinic (78, 79). Ideally, studies into site-specific TME are best performed in human tissue, but preclinical models may still provide key insights into this complex problem.

In summary, despite the challenges in investigating tissue-specific TMEs, a thorough understanding should take priority to improve the success of both current and future immunotherapies. Increased effort in preclinical and clinical studies will assist in selection of future immunotherapy combinations according to the likelihood of therapeutic response in the tumor site. We propose that personalized immunotherapy should not only be individualized to the tumor but account for the differences in tissue-specific TME.

## Author Contributions

Conception and design: AO, MK, and CS. Write, review, and revision of the manuscript: AO, PL, AU, SL, PD, MK, and CS. Supervision: PD, MK, and CS.

## Conflict of Interest Statement

The authors declare that the research was conducted in the absence of any commercial or financial relationships that could be construed as a potential conflict of interest. The handling editor declared a shared affiliation, though no other collaboration, with the authors.
